# Primary Squamous Cell Carcinoma of the Renal Pelvis: A Case Report Series

**DOI:** 10.7759/cureus.60568

**Published:** 2024-05-18

**Authors:** Prathyaksha Variar, Aroonima Misra, Fouzia Siraj

**Affiliations:** 1 Department of Pathology, Indian Council of Medical Research (ICMR) National Institute of Pathology, New Delhi, IND

**Keywords:** renal oncology, nephrolithiasis, kidney pathology, renal pelvis tumor, squamous cell carcinoma (scc)

## Abstract

Primary squamous cell carcinoma (SCC) of the renal pelvis is one of the extremely rare tumors encountered in the kidney. It poses a diagnostic challenge for both the clinician and pathologist alike due to the sheer rarity of its occurrence and the multitude nature of its clinical presentation. A review of the literature over the last few decades shows just a countable number of cases documented, each bearing the testimony of the aggressive nature of this subtype. We hereby report three cases of SCC of the renal pelvis origin received at a tertiary care hospital in North India.

## Introduction

Squamous cell carcinoma (SCC) of the kidney arising in the renal pelvis is unique and exceptional in its own way owing to the fact that it accounts for only <0.5%-0.8% of the reported malignant renal tumors [1]. It is often seen associated with long-standing cases of nephrolithiasis with recurrent retrograde infection and chronic inflammation of the pelvicalyceal lining [1-3]. These tumors are often found extending into the adjoining the renal parenchyma, ureter, and hilar fat. We hereby report three cases of this malignancy received for histopathological and immunohistochemical evaluation.

## Case presentation

Case 1

Clinical Presentation 

A 40-year-old female presented with abdominal pain and fever for two months. The pain was in the right hypochondrium and lumbar region. It was dull with no radiation. The fever was low grade and intermittent. There was no other significant past medical and family history. On general physical examination, the patient had severe cachexia with clinically evident pallor. Examination of the abdomen revealed a mass in the right lumbar region measuring 3 x 3 cm which was lobular, nontender, and moved with respiration. Renal angles were nontender.

**Figure 1:**
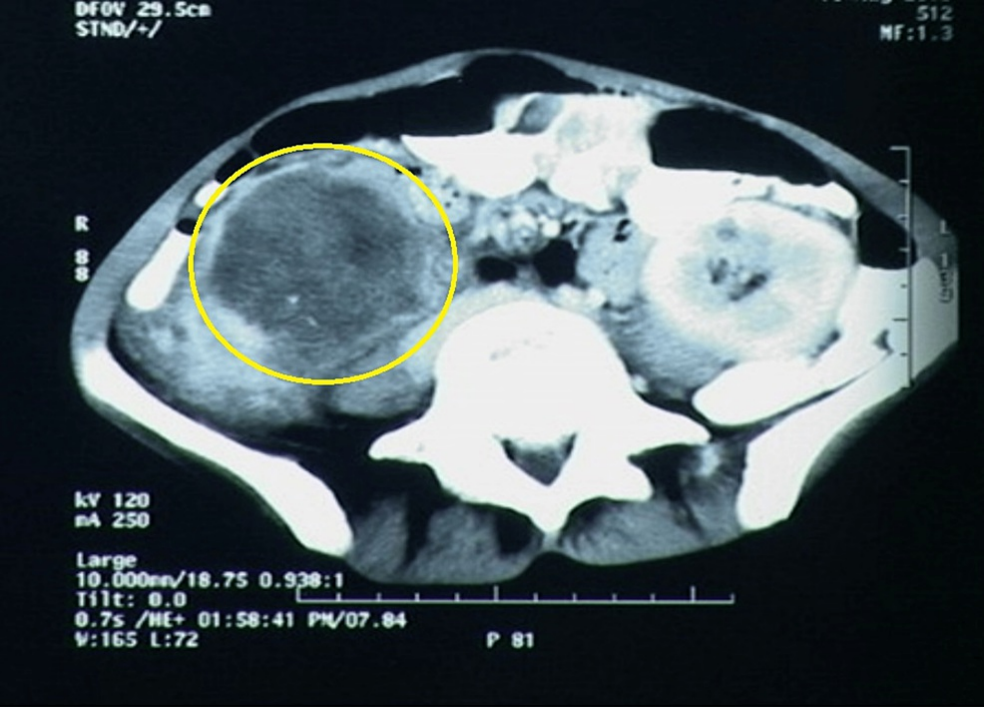
Lesion as observed on the axial section of the CECT scan abdomen showing an irregular hypodense mass lesion (yellow circle) measuring 87 x 67 mm in the axial plane in the mid and lower pole of the right kidney having internal calcific foci and few renal calculi occupying the renal pelvis CECT: Contrast-enhanced computed tomography

Laboratory investigations revealed hemoglobin of 5.4g/dl. The rest of the biochemical investigations including urine examination were within normal limits. Ultrasound of the whole abdomen revealed right pyonephrosis with calculus in the renal pelvis and multiple paraaortic lymph nodes. Contrast-enhanced computed tomography (CECT) scan was performed (Figure 1) which revealed an ill-defined mass involving the middle and lower pole of the right kidney with multiple calculi in the pelvis ranging from 1 mm to 5 mm. The left kidney appeared unremarkable. A lesion measuring 4.2 x 3.7 cm was also noted in the left lobe of the liver. The possibility of a metastatic renal malignancy in the background of nephrolithiasis was suggested in the right kidney. At this stage, differential diagnosis included a renal cell carcinoma variant (like sarcomatoid) and urothelial cell carcinoma.

However, when CT-guided fine-needle aspiration cytology (FNAC) was performed on the liver lesion, large atypical cells were seen arranged cohesively in sheets with dirty necrotic background and few intervening capillaries. These cells showed hyperchromatic nuclei with irregular nuclear membrane, abundant cyanophilic cytoplasm with an occasional orangeophilic cell. Surprisingly enough, cytomorphological features seemed to strongly favor the possibility of a metastatic SCC.

**Figure 2:**
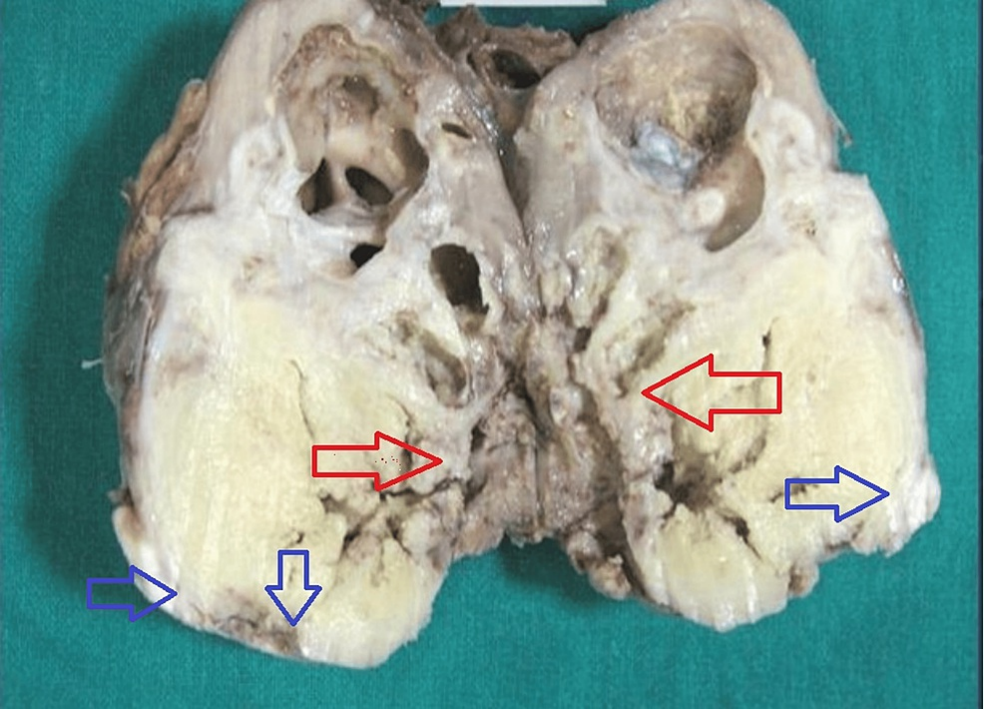
Gross appearance of the resected radical nephrectomy specimen showing a grossly dilated pelvicalyceal system with a large firm, yellowish-white tumor involving the middle and lower pole extending into the sinus fat (red arrow) with extensive areas of necrosis. Capsular breach is noted at one end (blue arrow)

Subsequently, a right open radical nephrectomy was performed. Intraoperatively, a huge renal mass measuring approximately 9 x 8 cm (Figure 2) was seen which was adherent to the renal vessels. Multiple lymph nodes at the hilum and paracaval region were identified. Colonic segment was adherent to the lower pole of the kidney with dense adhesions. The liver also showed multiple nodular lesions.

The postoperative course of the patient was stormy due to an anastomotic leak from the colon for which she underwent another surgery on the third postop day. On the eighth postoperative day, the patient succumbed to the disease and expired.

Pathological Findings

Grossly, a right radical nephrectomy specimen measuring 10.5 x 9.6 x 7.2 cm was received. The outer surface was bosselated with capsular breach noted (Gerota's fascia uninvolved). The cut section revealed a large tumor mass in the mid and lower pole measuring 8.7 x 7.5 cm extending into the pelvis and sinus fat. The cut surface was grey white, firm with areas of extensive necrosis. In the upper pole, the calyces were markedly dilated with a thin rim of renal parenchyma in the periphery. Multiple calculi were seen with the largest one measuring 3 cm in diameter lodged in calyces. Multiple lymph nodes were isolated ranging in size from 0.5 cm to largest measuring 1.1 x 0.8 cm.

Microscopic Findings

**Figure 3:**
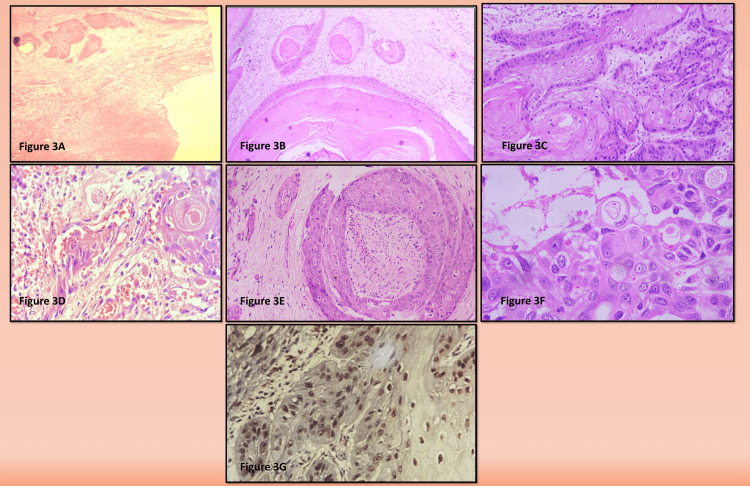
(A) Low-power view (H&E stained, 4x) showing tumor nests of squamoid origin adjacent to the renal pelvis; (B) H&E stained, 10x showing areas of the renal pelvis with tumor nests of squamoid origin; (C) H&E stained, 10x view showing the nests of tumor cells with keratin pearl formation; (D) H&E stained, 40x view showing few small tumor nests exhibiting lymphovascular invasion; (E) H&E stained, 10x view showing few small tumor nests exhibiting perineural invasion; (F) H&E stained, 40x view showing few plump spindle-shaped tumor cells exhibiting sarcomatoid morphology; (G) Immunohistochemical stain demonstrating a strong nuclear p63 positivity in tumor cells H&E: Hematoxylin and eosin stained

Microscopic examination from multiple sections of the renal pelvis revealed an ulceration of the epithelium with the submucosa showing dense inflammatory infiltrate comprising sheets of foamy histiocytes, lymphocytes, plasma cells, and giant cells. Sections from the mass (Figures 3A-3C) revealed tumor cells arranged predominantly in nests, islands, and single cells involving the pelvicalyceal lining and renal parenchyma with capsular breach noted focally. Cells were large, round to polygonal shape with markedly pleomorphic vesicular nuclei containing prominent nucleoli. Few areas showed spindling of the tumor cells. Abundant keratin pearl and squame formation was seen. Lymphovascular invasion and perineural invasion (Figures 3D-3E) were also noted. On immunohistochemistry tumor cells showed strong nuclear positivity for p63 (Figure 3F) with patchy cytoplasmic positivity for cytokeratin 7 (CK7). Out of six lymph nodes isolated, one node showed tumor deposit with no extranodal extension. A final diagnosis of moderately differentiated primary SCC of the right renal pelvis, pT3N1M1 with nephrolithiasis, and xanthogranulomatous pyelonephritis was made.

Case 2

Clinical Presentation

A 73-year-old male presented with low-grade intermittent fever for two months, not associated with rigors/chills. The patient also complained of increased frequency of micturition but no dysuria or hematuria. There was a significant weight loss in the last two months. The patient reported a history of nephrolithiasis in the past which spontaneously resolved with medical management. No other significant medical and family history were given. General physical and systemic examination including per abdomen palpation was unremarkable.

**Figure 4:**
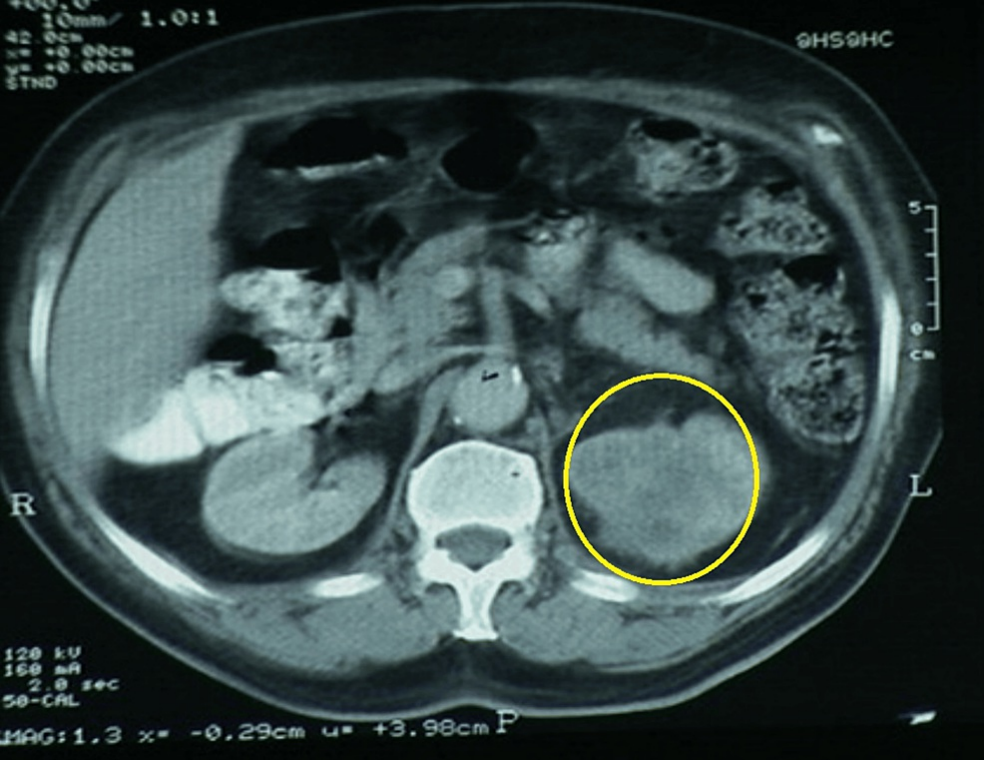
Axial section of the CECT abdomen showing a heterogeneously enhancing mass measuring 54 x 42 mm (marked in yellow circle) in the superior pole of the left kidney

Laboratory investigations revealed that mild anemia and urine examination showed few white blood cells (WBCs) (6-8/hpf). Ultrasonography (USG) abdomen revealed a well-defined hypoechoic mass lesion measuring 5.2 x 4.5 cm in the superior pole of the left kidney. CECT abdomen showing a heterogeneously enhancing mass measuring 54 x 42 mm was noted in the superior pole of the left kidney (Figure 4). Differential diagnosis at this stage included xanthogranulomatous pyelonephritis, renal cell carcinoma variant (like sarcomatoid), or urothelial cell carcinoma. Diethylenetriamine pentaacetate (DTPA) scan showed impaired left renal function, while the right kidney revealed normal function. 

The patient underwent left open radical nephrectomy. Intraoperatively a renal mass arising from the upper and mid pole was seen measuring 7 x 5 cm. A lymph nodal mass was seen at the hilum which was adherent to aorta.

The patient's immediate postoperative period was uneventful. No adjuvant therapy was administered during the three-month postoperative period after which the patient was lost to follow-up despite repeated efforts of establishing contact.

Pathological Findings

**Figure 5:**
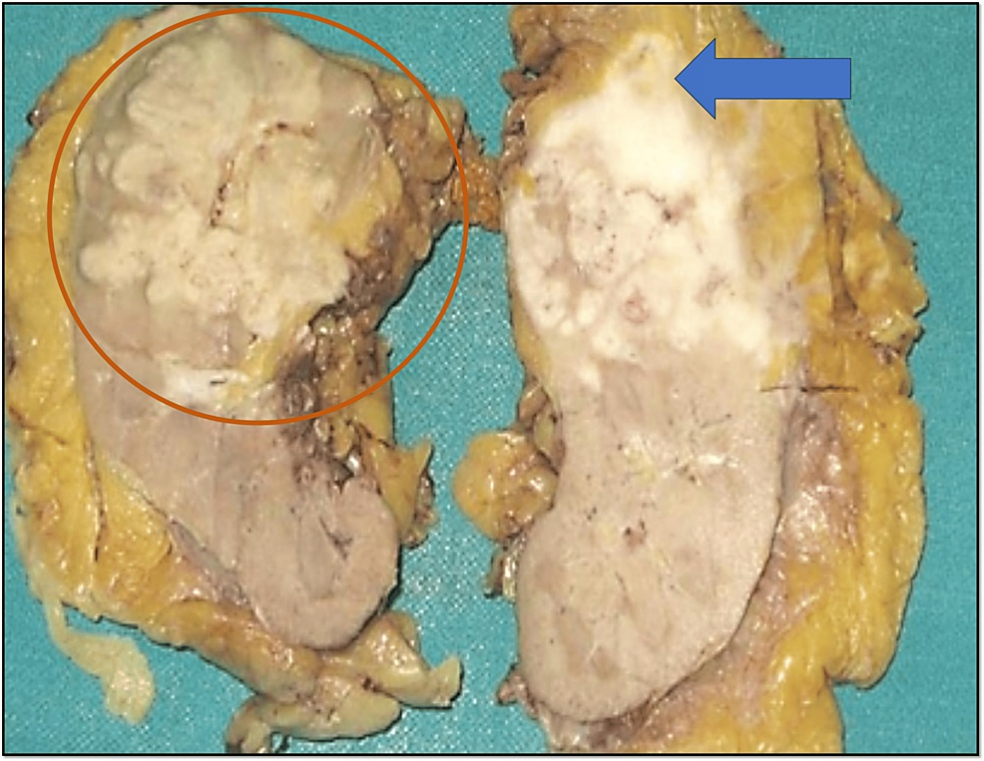
Gross appearance of the resected cut-open radical nephrectomy specimen showing greyish white, firm, and lobulated tumor (marked in red circle) involving the renal pelvis and upper pole extending into the perinephric fat (marked by blue arrow) and adjoining adrenal gland

Grossly left radical nephrectomy specimen (Figure 5) measured 12 x 8 x 6 cm. Sections revealed an ill-defined tumor in the upper and middle poles measuring 6 x 6 x 5.5 cm. The tumor was infiltrating the capsule and extending into perirenal fat which on further sectioning was noted involving adrenal gland. The cut surface was greyish white, firm, and lobulated with areas of necrosis. The tumor was also infiltrating the renal pelvis. The rest of the kidney showed normal corticomedullary demarcation. Five lymph nodes were isolated from submitted lymph node mass out of which three were matted and filled with necrotic material.

Microscopic Findings

Microscopic examination showed a malignant tumor composed of nests, sheets, and whorls of squamous cells with keratin pearl formation. Tumor cells involved pelvicalyceal lining with infiltration seen into the renal parenchyma and capsule of the kidney into the surrounding perinephric fat. It was also extending into the pelvis; however, the ureter was spared. Perineural and lymphovascular tumor infiltration was present. The adrenal showed a small focal infiltration by tumor nests. Immunohistochemistry performed for p63 and CK7 showed a similar pattern of expression in tumor cells as discussed previously. Also out of the five lymph nodes examined, three lymph nodes appeared matted and showed metastatic deposits. A final diagnosis of moderately differentiated primary SCC of the renal pelvis, left kidney, and pT4N1M0 was made.

Case 3

Clinical Presentation

A 47-year-old male with long standing history of renal calculi presented to the outpatient department (OPD) with dull-aching, right flank pain for six months and hematuria since one week. The pain was initially episodic, but subsequently became persistent, low grade, and dull aching. The clinical examination was unremarkable.

Laboratory investigations were within normal limits except for a few pus cells in the urine examination. Radiological evidence was suggestive of an enlarged kidney with multiple renal calculi and dilated pelvicalyceal system with a well-defined hypoechoic lesion seen in the renal pelvis measuring 5.5 x 3.3 cm. Few enlarged hilar lymph nodes with no evidence of systemic metastasis were seen. DTPA scan was suggestive of impaired renal function of the right kidney with normal left kidney. Differential diagnosis at this stage included xanthogranulomatous pyelonephritis, renal cell carcinoma variants, and urothelial cell carcinoma.

The patient underwent right laparoscopic nephrectomy wherein a grossly enlarged kidney was found with almost 100 ml purulent-exudate aspirated from its lower pole. Also seen was a renal mass arising in the middle pole from the pelvicalyceal system measuring 5.7 x 3.5 cm interspersed by multiple calculi.

The patient's postoperative period was uneventful. No adjuvant therapy was given to the patient. He was disease-free during the follow-up period of around eight months after which patient was lost to follow-up.

Pathological Findings

Grossly nephrectomy specimen measured 12.5 x 8.5 x 5.0 cm. Sections revealed few renal calculi (0.5-3 mm) with an ill-defined tumor in the middle pole measuring 5.8 x 4.5 x 3.5 cm. The cut surface was greyish white, firm, and lobulated with patchy areas of necrosis. The tumor was abutting the renal capsule but extending into the sinus fat. The rest of the renal parenchyma showed fibrotic changes with loss of corticomedullary differentiation. Adrenal gland and ureter were spared. Three subcentimetric lymph nodes were isolated from the submitted lymph node mass.

Microscopic Findings

**Figure 6:**
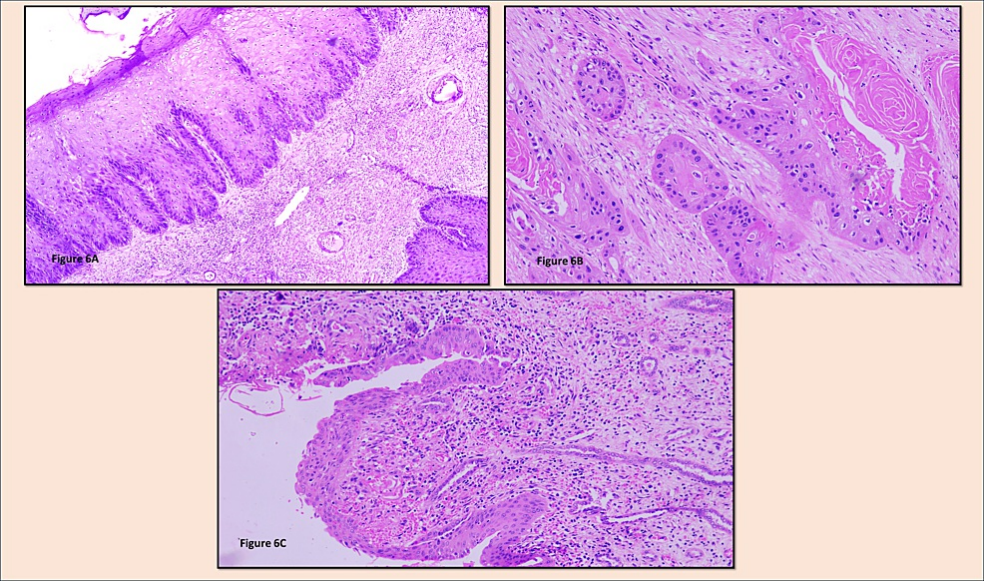
(A) H&E stained, 10x view showing extensive squamous metaplasia along the renal pelvis lining; (B) H&E stained, 10x view showing well-formed tumor nests with keratin pearl deposits in the renal parenchyma; (C) Adjacent renal parenchyma showed compressed tubules with chronic pyelonephritis changes

The pelvic lining showed squamous metaplasia with multiple nests of tumor cells of squamoid origin arising and infiltrating into the renal parenchyma involving sinus fat. Adjoining parenchyma showed glomerulosclerosis with thyroidization of tubules and marked lymphocytic infiltrate (Figures 6A-6C). No features of lymphovascular/perineural invasion were seen. Out of three lymph nodes isolated, all were free of tumor. A final diagnosis of moderately differentiated primary SCC of the right renal pelvis (pT3a) with chronic pyelonephritis was made.

## Discussion

SCC of the kidney constitutes 0.5%-0.8% of malignant renal tumors and 4% of upper urinary system tumors [1]. Usually occurring in fifth to seventh decade, they are believed to arise from the undifferentiated stem cells of the renal origin [2]. Although the exact pathogenesis of SCC arising natively in the kidney is unknown, few studies postulate its origin from squamous metaplasia of the urothelium. The most commonly documented risk factors in literature include long-standing cases of nephrolithiasis, recurrent or poorly treated urinary tract infections, and exposure to endogenous and exogenous chemicals [3]. Rare case reports of vitamin A deficiency, radiotherapy-associated disease with few tropical parasitic infections like schistosomiasis have also been reported [4]. These causative agents often lead to chronic irritation of the urothelial lining which may become a prelude to the development of leukoplakia. These changes may further evolve to undergo neoplastic transformation over a period of time, thus giving rise to this uncommon malignancy of the renal pelvis [5].

The clinical presentation of the patient is often nonspecific. Patients usually present with insidious onset dull atypical flank pain and hematuria with occasional febrile episodes [2,5] as was seen in our cases. Imaging modalities including USG and CECT/18-Fluoro-deoxyglucose (FDG)-positron emission tomography often show features suggestive of hydronephrosis with mass lesions involving pelvicalyces with extension into the renal parenchyma. These findings, although provide a valuable aid in disease localization and staging, are nonspecific as they can be commonly seen in vast spectrum of differentials including benign xanthogranulomatous pyelonephritis to other renal malignancies [6]. 

Thus, the mainstay of diagnosis is essentially based on microscopic confirmation. However, challenges do not end here as due to the rarity of its occurrence in the kidney, a dilemma often faced by the diagnosing pathologist in such a situation is to ascertain whether to categorize the renal mass as primary renal SCC, extensive squamoid differentiation in urothelial carcinoma of pelvis, or SCC metastasis to kidney [7]. In the presence of coexisting urothelial dysplastic lesions including urothelial carcinoma in situ (CIS), the tumor should be diagnosed as urothelial carcinoma with squamous differentiation. Therefore, the urothelium of the renal pelvis should be histologically normal for the diagnosis of primary SCC of the kidney parenchyma.

Although there are no pathognomic signs specific to the entity, certain symptoms associated with paraneoplastic symptoms like hypercalcemia and thrombocytosis [8] besides regional metastasis have been occasionally reported with advanced disease pathology.

Owing to the late clinical presentation, case detection often occurs at the locally advanced or metastatic stage. There is a lack of standardized treatment protocol for the management of patients with primary renal SCC. Surgical approach including radical nephrectomy or nephroureterectomy with regional lymph node dissection, combined with adjuvant chemotherapy and/or radiotherapy, has been tried with limited success rates. Trials with cisplatin-based adjuvant chemotherapy with or without radiotherapy have been conducted; however, not much survival benefit has been demonstrated. Thus, although such integrated approaches help in tumor debulking to an extent, overall patient mortality rate has not been encouraging with few studies suggesting survival rates even less than one year from the time of diagnosis and five-year survival rates as low as 10% [9].

## Conclusions

Primary SCCs of the renal pelvis are rare renal tumors with an aggressive clinical course. Much of the lacunae in treatment arise due to the late detection of the disease. The crucial time period for instituting curative treatment is lost which has a detrimental effect on the overall five-year survival rate of the patient.

Hence, a strong clinical index of suspicion must be kept for this entity, especially when dealing with renal mass lesions in long-standing cases of nephrolithiasis and recurrent urinary tract infections. A combined approach using radiological findings and histopathological confirmation is warranted for diagnosis. This will ensure an early detection and timely staging of the disease, which in turn offers a silver lining for improving the current case survival statistics. Also considering the limited literature documented about this subtype, we recommend the need to expand further clinical and molecular studies in this area to widen the clinician's treatment armamentarium.
